# Comparing Macroscopic and Quantitative Histological Methods to Determine Sexual Maturity in the Female European Plaice, *Pleuronectes platessa* Linnaeus, 1758

**DOI:** 10.3390/ani16030519

**Published:** 2026-02-06

**Authors:** Carine Sauger, Jérôme Quinquis, Kristell Kellner, Clothilde Berthelin, Mélanie Lepoittevin, Nicolas Elie, Laurent Dubroca

**Affiliations:** 1 Laboratoire Ressources Halieutiques de Port en Bessin, Institut Français de Recherche pour l’Exploitation de la Mer (IFREMER), Avenue du Général de Gaulle, 14520 Port en Bessin Huppain, Francelaurent.dubroca@ifremer.fr (L.D.); 2 Marine Ecosystems and Organisms Reasearch Lab, Université de Caen Normandie, Normandie Université, MERSEA UR 7482, Esplanade de la Paix, 14000 Caen, France; kristell.kellner@unicaen.fr (K.K.); clothilde.berthelin@unicaen.fr (C.B.); melanie.lepoittevin@unicaen.fr (M.L.); 3 VIRTUAL’HIS, Service Unit PLATON, Federative Structure 4207 “Normandie Oncologie”, Université de Caen Normandie, Normandie Université, Esplanade de la Paix, 14000 Caen, France; nicolas.elie@unicaen.fr

**Keywords:** maturity determination, stereology, human assessment, inter-rater reliability, histology, oogenesis

## Abstract

Two methods (macroscopic and quantitative histology) to determine sexual maturity are described in this paper, with an emphasis on the methods used in stereology. Based on a point counting grid (Glagolev’s method), ovarian cellular structures were quantified throughout the whole cross section of 151 *Pleuronectes platessa* individuals. The novelty of this paper is checking the cellular homogeneity in the anterior, median and posterior sections of both ovaries from 15 fish, accompanied by a reading protocol to limit subjectivity during stereology readings. Once the ovarian structures were quantified for 151 individuals, a model was set up following the standardized staging grid of the International Council for the Exploration of the Sea (ICES), classifying each sampled individual into a maturity phase. These results showed that 23.4% of the time immature (A) individuals were correctly identified through the macroscopic method, but 47.9% of developing (B) individuals were classified under A, while individuals that had finished spawning were correctly identified as such 80.4% of the time. Finally, the maturity ogive and the length at which 50% of the population is sexually mature (L_50_) were calculated, with an L_50_ of 28.6 cm when using the macroscopic method and an L_50_ of 20.6 cm with the stereological method.

## Simple Summary

To implement regulations in fisheries, like the minimum catch size for a species, data on the species’ biological parameters must be collected and analyzed. Among these parameters, sexual maturity of the fish is evaluated. Multiple methods exist, and in this paper we used the macroscopic method (visual appreciation of the sexual organ to classify the fish into a maturity phase) and the stereological method (identifying and counting cells inside the fish’s sexual organs to determine the maturity phase). The methods put forward try to minimize the subjectivity brought on by the human agent that will determine the maturity phase, through the implementation of guidelines and calibration exercises. Beyond lowering the human assessment bias, this article also shows that the macroscopic method, used to collect data that will later be exploited to set up fishery regulations, accurately determines the sexual maturity of female plaice only 40.4% of the time. This leads to estimations of size at first maturity of 28.6 cm for the macroscopic method compared to 20.6 cm when using stereology, differences in sizes that could have a major impact when setting up fishery regulations.

## 1. Introduction

Maturity is an important life history trait that defines a population’s dynamics [1] and can be used to quantify the reproductive capacity of individual fish [2]. In fisheries’ stock assessments, demographic models are based on length composition from commercial catches and fishing surveys [1,3]. In this regard, an accurate determination of the length or age at which a commercial stock species has reached sexual maturity ensures a precise appraisal of its size and reproductive capacity [4,5]. Moreover, factors such as the maturity ogive [3,6] and the length at which 50% of the population has reached sexual maturity (L_50_) [7] are extracted from maturity data and are used in stock assessment models. This makes maturity data basic information for estimating the number of mature individuals within a stock, also known as the Spawning Stock Biomass (SSB) [8]. The SSB is an important variable in fisheries management [9] for it is used in population dynamic models and when estimating the Total Allowable Catch (TAC) for stock species from one year to the next [3,8].

For maturity determination, multiple maturity scales and terminologies can be found throughout the literature when classifying the ichthyological reproductive cycle [10,11,12,13,14,15,16]. Moreover, these maturity scales are in constant evolution and will differ from one institution to another [11]. Since the early 2000s, the International Council for the Exploration of the Sea (ICES) has worked towards harmonizing the terminologies and scales used within different European institutions in order to better homogenize these practices [11,12,17,18,19,20,21,22]. The most common method used to determine sexual maturity in fish is the visual observation of the gonads [6,11,23] (i.e., the macroscopic method). Unfortunately, this simple and swift method leads to uncertainties in the identification of the maturity phase through the use of subjective criteria such as the texture, color or size of the gonads [23]. Disagreements between maturity estimators are a common problem, with higher uncertainties that may even be found when estimating immature individuals and individuals that have reached first maturity [19,24].

In this study, the plaice (*Pleuronectes platessa*, Linné 1758) was chosen as a model species to address this issue. The plaice is a well-studied commercial species that ranges from the west of the Mediterranean Sea, along the European coast, and reaches the Icelandic waters while passing through the North Sea and the Skagerrak [25,26,27]. This teleost is a synchronous batch spawner [13,28], meaning that females will spawn a portion of their eggs at multiple intervals in the spawning season [10]. The spawning season for the plaice spans from December to March [3,29,30,31], with females reaching sexual maturity at 4 or 5 years of age [26,27]. During macroscopic sexual determination for plaice, ref. [23] found that the percentage agreement between readers ranged from 40% to 94%, depending on the maturity phase. Moreover, a workshop organized by the International Council for the Exploration of the Sea (ICES) [21] estimated that the overall agreement for plaice maturity staging was 80%, with a percentage agreement between readers that varied from 67% to 83% depending on the maturity phase. Only female gonads were analyzed, with female gametes being easier to observe through their larger size compared to male gametes, as well as bringing information on egg production for reproductive capacity estimations in stock assessments [10].

To provide a more accurate identification of an individual’s maturity phase, methods like histology and whole mounts may be used [1], with histology being the more precise method that also yields more information [32]. This led to the use of histology on an individual’s gonads to classify the sampled fish within a maturity phase [10,32]. In our case, histology and stereology were used to quantify the different cellular structures found within plaice’s ovaries, with stereology defined as “a body of mathematical methods which relate parameters defining three-dimensional structures to measurements obtainable on two-dimensional sections” [33]. Even though histology takes out a lot of the subjectivity during the maturity determination process, it is not perfect. First and foremost, the maturity phase is classically determined by the most advanced germinal cell present on the slide, thus relying on the reader’s capacity to spot and identify the cellular structure. Secondly, there is the issue of whether cell development is homogeneous throughout the entire gonad, and between gonads for the same individual. If that is not the case, then there is a risk of obtaining different results depending on the location of the histological cross section. Finally, to properly identify certain maturity phases or cellular development dynamics from histological slides, applying a quantitative method is mandatory.

**Figure 1:**
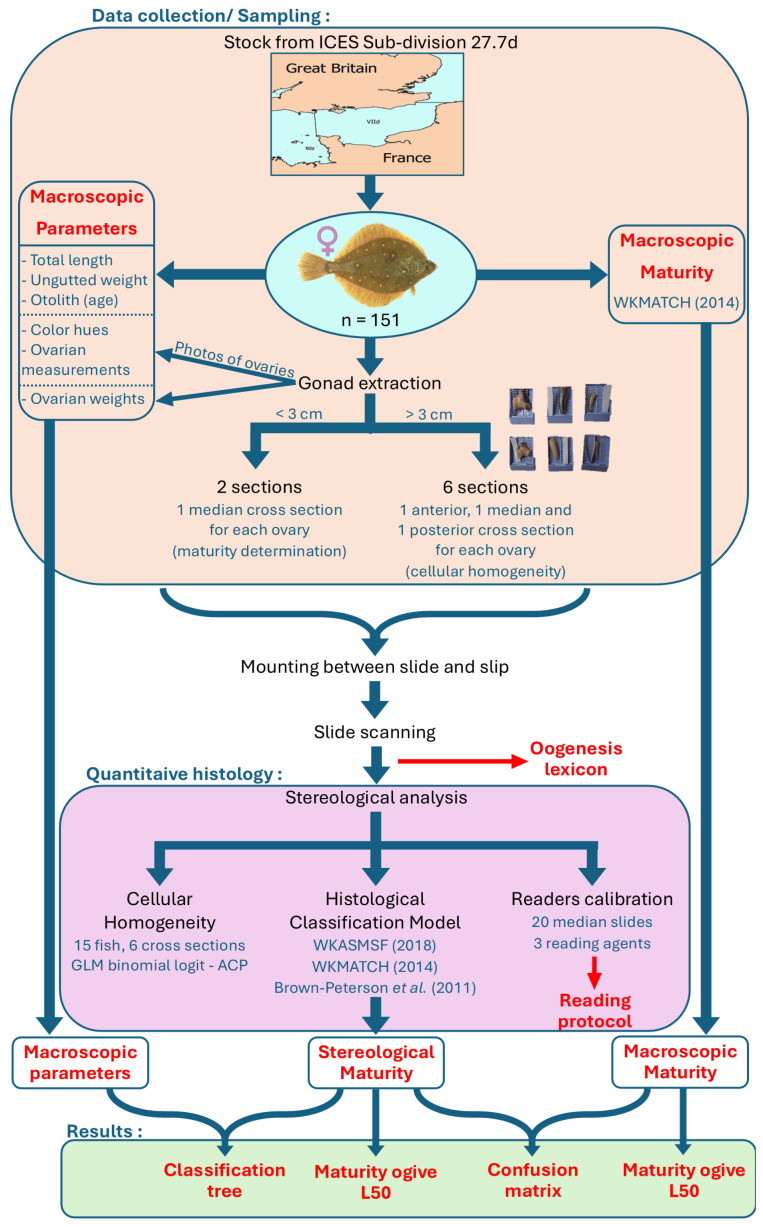
Summary of the material and methods with the used references [10,11,12], as well as result outputs for the current study.

A detailed breakdown of the methods applied in this study has been published in a data paper describing the data set used [34]. From the same data, the ovarian germline cells were presented in [35], as well as results such as size at first maturity (Lp_50_), an overview of the oogenesis cycle through time, and an appreciation of the volumes each germline cell takes depending on the maturity phase of the individuals. The individuals’ maturity phases obtained with the macroscopic method were used to underline that there was a classification problem when cellular data was added to validate the maturity phase. The following study goes more in-depth with the use of these methods (Figure 1). First of all, multiple biological parameters, that are not classically measured, were taken (gonad lengths, gonad color hues) to test if, once a reliable maturity phase was determined, one or more of these parameters could be used to better assess the maturity phase with the macroscopic method through the use of a classification tree.

Secondly, in the present study a major emphasis was put into the methods accompanying quantitative histology (stereology), starting with calibration exercises between different agents to assure the reproducibility of the stereology reading process. To help with this endeavor, a lexicon [36] describing different cellular structures, as well as an identification protocol [37], were set up to minimize the reading bias between operators. Cellular homogeneity was verified between and within the gonads of the same individual, with the use of a general linear model on the cell counts from cross sections taken at different areas of the same gonad. Finally, a maturity phase classification model was set up to classify individuals into a maturity phase using the stereological data. For this, the definitions and staging grids of Brown-Peterson et al. [10] and the ICES (WKASMSF [11], WKMATCH [12]) were used. Once the individuals were categorized into a maturity phase, maturity phase results obtained through macroscopic readings and through the stereological method were compared, as well as their respective L_50_.

## 2. Materials and Methods

### 2.1. Sampling

A total of 151 female plaice were collected by bottom trawling in the English Channel (ICES division VIId). The specimens were gathered during 10 different sampling events, from January 2017 to August 2019 (Table 1). Each fish was measured (total length in cm), weighed (ungutted weight in grams) and aged through otolithometry. An experienced operator estimated each female’s sexual maturity with the naked eye while following the maturity staging grids of the ICES [12].

### 2.2. Full Ovarian Photographs

Both ovaries were extracted and images were captured using a Nikon D3200 digital camera (Nikon Corporation, Tokyo, Japan). The ovaries were positioned onto a uniform colored background, next to a €0.50 coin that served as a fixed-size marker. The Image J software (v. 1.50J [38]) was used to analyze the photographs and quantify the color hues, as well as the macroscopic parameters, of each ovary: surface (mm^2^), length (mm), width (mm), width at mid-length (mm). Photographs can be found under the Zenodo repository [39].

### 2.3. Gonad Extraction, Fixation and Mount Between Slide and Slip

Ovaries were cut and placed into tissue processing embedding cassettes. For ovaries of 3 cm and over, the dorsal ovary (coded D) and ventral ovary (coded V) were cut into 3 sections of 1 cm. These sections were located in the anterior (coded 1), median (coded 2), and posterior (coded 3) areas of each ovary. Each sample was placed into an embedding cassette, with an identification tag.

The tissue processing embedding cassettes were placed for 48 h into a Davidson solution for tissue fixation, before being placed into an automate (Leica TP1020, Leica Biosystems, Nussloch, Germany) for dehydration and paraffin embedding. The ovarian samples were then cut into 5-micron-thick sections, using a microtome (Leica HM330, Leica Biosystems, Nussloch, Germany). These sections were placed onto a slide, deparaffinized, rehydrated, stained in Prenant-Gabe’s Trichrome [40] and mounted with Roti-Histokitt^®^, Carl ROTH, Karlsruhe, Germany.

### 2.4. Slide Scanning and Stereological Readings

Each slide was digitized using a histology slide scanner, Aperio CS, running under the Scan Scope Console software (v.10.2.0.2352, Leica Biosystems, Nussloch, Germany), with a magnification of 20× (numerical aperture 0.75). The Aperio software (v12.1.0.5029 [41]) assisted with the counting of the 19 different types of cellular structures found within the ovarian scans, using stereological analysis based on Glagolev’s method [42], an assumption-based stereological method that uses a grid of points to estimate the different structures’ areas on the total amount of points sampled. The sampling grid was composed of 500 to 600 sampling points equidistant from one another, ensuring the same sampling effort for every slide (see [34] for further details). The counted percentage of each structure (**fract_estim**) was then calculated.

fract_estim=(100/total_points)∗hit_points

with **fract_estim** the percentage (%) of times the structure was counted, **total_points** the total number of sampling points counted during the stereological analysis on the histological slide, and **hit_points** the number of times a structure was counted on the histological slide.

The 19 structures identified within the plaice’s oogenesis cycle were described in a lexicon [36] as well as in a journal article [35]. The Table 2 summarizes the terms and abbreviations used in the two previously stated papers, and were used for the current study. A total of 226 histological slides were read using the aforementioned stereology method, with 151 slides of the median section of the ventral ovary (V2) read for maturity staging. Prior to the reading of the 226 slides, 20 slides of the median section of the ventral ovary (V2) were used for the agent calibration exercise.

The calibration exercise consisted in having three agents read the exact same 15 slides and apply a reading error index for each cellular structure. If a structure showed a difference of over 3% between the readers, the slides were reviewed and identification rules were set up under a reading protocol [37]. In this aforementioned reading protocol, rules were set up to guide the readers on how to proceed depending on where the point of the grid lands within the histological section. Once these guidelines were acquired, the previous 15 slides were then read a second time by the same three agents. The results from both readings of the calibration exercise led to the quantification of a reading disagreement percentage between the three agents for each slide through the use of two inter-rater reliability indexes: the percentage agreement between readers [43], and Fleiss’s kappa [44,45].

### 2.5. Cellular Homogeneity

For a detailed view of the analyses of this section, refer to the Supplementary Material section.

To assess cellular homogeneity within the ovary and between the ventral and dorsal ovaries, 6 slides matching the anterior, median and posterior sections of the dorsal and ventral ovaries, respectively coded D1, D2, D3 and V1, V2, V3, were read. The 30 sampled ovaries did not show oocytes in late vitellogenesis (**vit3**), hydrating oocytes (**pho**) or hydrated oocytes (**ho**).

All computations were performed under RStudio (v1.2.5001 [46]). Once all 90 slides were read, the reading error index (in %) was calculated for each type of cellular structure. For all 15 individuals, histograms with the number of times each cellular structure was counted were established to better visualize the results. Afterwards, general linear models (GLM) were performed to check the effects of the section position within the gonad, as well as cellular structure occurrences within these sections. The response variable used was the number of times a structure was counted on a single slide divided by the total number of sampled points on that same slide. The error term followed a binomial distribution, and a logit regression model was used [47,48]. The model results were then analyzed by using the deviances of each variable (the 19 cellular structures). The function *drop1* [47] was used to quantify the deviances of each variable by removing them from the whole model alternatively. A principal component analysis (PCA) on the histological structures was established to summarize and plot the reading data.

### 2.6. Maturity Phase Determination

**Figure 2:**
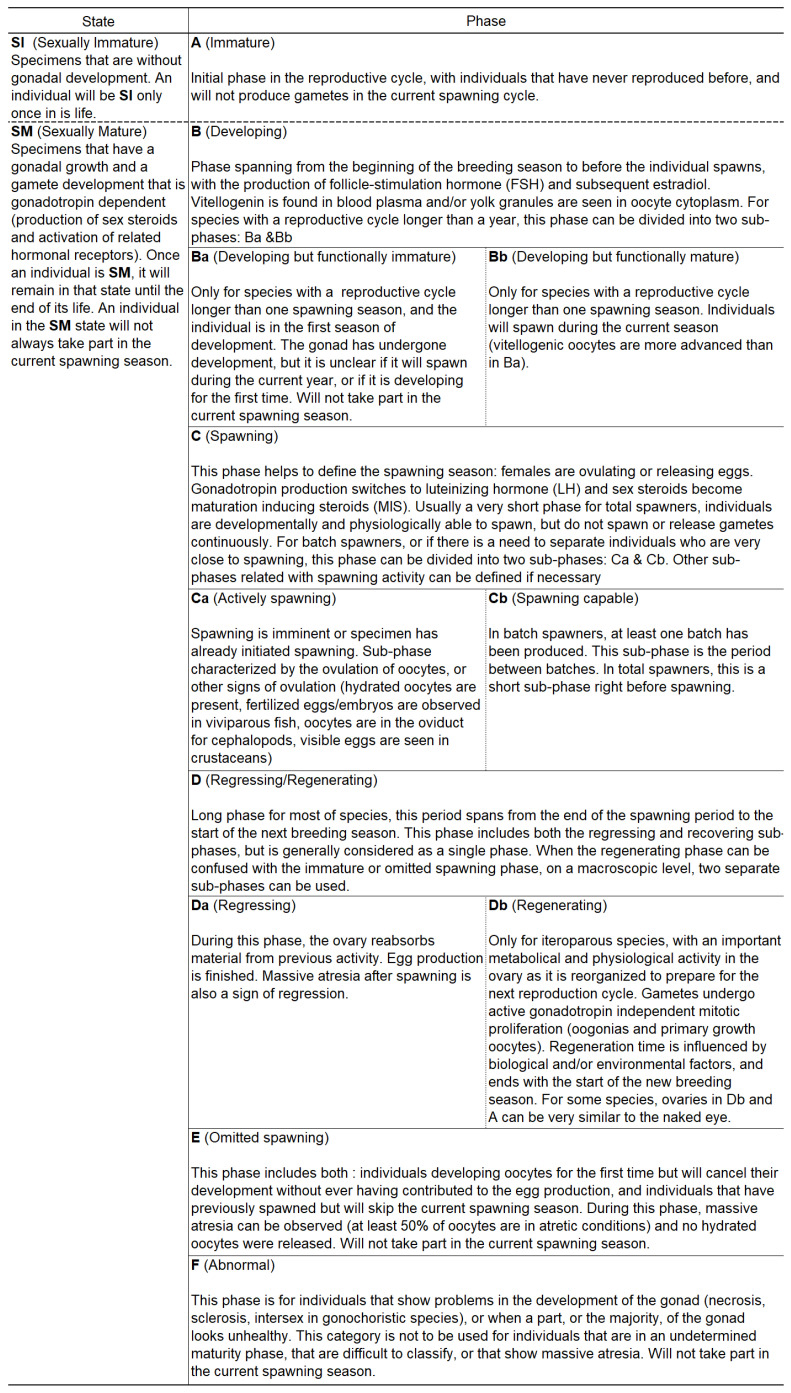
Definitions for the sexual states and maturity phases in teleosts, established from the WKMATCH [12], WKASMSF [11] and Brown-Peterson et al. [10].

Following the ICES definitions and staging grids [11,12], as well as the terminologies from Brown-Peterson et al. [10], Figure 2 was established for gonochoristic oviparous female marine teleosts. From these documents, Table S2 was set up, summarizing macroscopic and microscopic ovarian criteria for the different maturity phases, as well as the stereological criteria used to classify the 151 sampled fish into the corresponding maturity phase. Using the stereological reading results of the median ovarian sections (V2), R Studio was used to classify the 151 sampled individuals into either the immature (A), developing (B), spawning (C) or omitted spawning (E) maturity phase. All remaining fish not classified into one of the 4 previously stated phases were put under the regressing/regenerating phase (D) as shown in Figure 2 (Histological model).

Once each fish was classified into a maturity phase, a confusion matrix between the maturity phase estimated through macroscopic criteria and the stereologically estimated maturity phase was established. Classification trees were set up using the stereologically determined maturity phases coupled with macroscopic parameters that could easily be measured or collected in the field: the month at which the fish was sampled (1 to 12 for the months going from January to December), total fish length (cm), ungutted fish weight (g), fish age (year), length of the ventral ovary (mm), width of the ventral ovary (mm), width at mid-length of the ventral ovary (mm) and the ratio between the ventral ovary’s length and the fish’s total length. Finally, the maturity ogive and the length at which 50% of the population has reached sexual maturity (L_50_) were calculated using the *sizeMat* package [49]. Maturity ogive and L_50_ were calculated from both the macroscopic maturity data and the stereological maturity data.

## 3. Results

### 3.1. Inter-Rater Reliability Percentage

The calibration exercise between three agents, for the stereology reading of 20 histological sections, was composed of multiple readings. Between the first and last reading, a stereology reading protocol [37] was set up so that all agents followed the same reading method. For the last reading, the percentage agreement between readers (Table 3) went from 69.0% to 87.8% for 12 slides, and the Fleiss’ kappa from 71.9% to 89.0% for 20 slides. The mean value for the percentage agreement was 79.2% (±6.1%), while Fleiss’ kappa was 81.2% (±4.7%). While the inter-rater reliability was estimated with a percentage agreement and Fleiss’ kappa for the last stereological slide reading, the first readings were not recorded, so the evolution of the reading method before and after the reading protocol was set up could not be compared.

### 3.2. Inter and Intra Gonad Homogeneity

**Figure 3:**
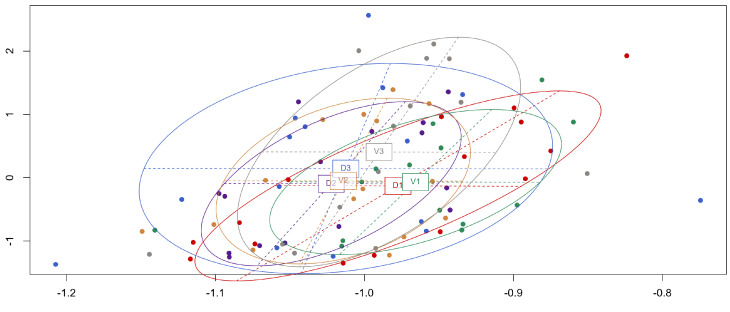
Principal component analysis (PCA) graphical output for the 90 histological slides read for *n* = 15 individuals, with the 6 different section positions color-coded (D1 red, D2 purple, D3 blue, V1 green, V2 peru, V3 gray) and 95.3% of the variation expressed on both axis.

Results of the *drop1* function (Table S1 in Supplementary Material) show the variance *slide* (identifying the sampled individuals) to always have the highest deviance, and thus best explains the models for all cellular structures. All *position* variables (position of the section within the gonads) have a lesser effect on our models than *slide*, even with very significant *p*-values (P(Chi) < 0.001). The presence of the cellular structure oogonium (**og**), does not seem significantly affected by the individual (*slide*) or the position for the section (*position*). However, the deviances are very low compared to the other models. This could be explained by the fact that these cellular structures are of very small size, and thus may be present on a slide but not recorded. The percentage of estimated **og** does not reflect reality. A principal component analysis (PCA) (Figure 3) simplified the data collected for each cellular structure and visually shows an overlap of ellipses for all slide positions, suggesting their similarity.

### 3.3. Maturity Phase Classification

To make use of the stereological method, counts of the cellular structures found within the ovaries were used to classify each fish into a maturity phase. The rules set to classify the fish into a maturity phase were based on the descriptions of the histological features of the ovaries from the WKMATCH [12], the WKASMSF [11] and Brown-Peterson et al. [10]. The rules set are as follows (in Supplementary Material in Table S2, last column):

For the immature (A) individuals, the histological features description states that only oogonia and primary growth oocytes are present, the ovarian wall is thin, and the connective tissue is scarce. To abide by those rules, individuals classified into this phase only showed oogonia (**og**), early-stage primary oocytes (**po1**) and late-stage primary oocytes (**po2**). All other follicle types can not be present on the cross section.

Individuals found in the developing stage (B) enter the sexually mature state. It is important to note that, by definition, an individual will leave the sexually immature state when cortical alveoli oocytes are produced [50]. In this regard, all ovarian cross sections must have follicles at the cortical alveoli stage, or follicles at later stages, in order to be classified into one of the phases found within the sexually mature state.

From a histological point of view, the oocytes will increase in size, blood vessels will become more distinct, and follicles from primary growth oocytes to the end of vitellogenesis (**vit3**) can be present, with at least cortical alveoli oocytes or later-staged follicles. Oocytes at the end of vitellogenesis (**vit3** and later) and post-ovulatory follicles (**POF**) must not be present. The model will classify individuals into the developing (B) phase when the following criteria are respected:

- Presence of either cortical alveoli oocytes (**cao**), oocytes in early vitellogenesis (**vit1**), oocytes with *zona radiata* growth (**vit2**).
- A count of alpha atretic oocytes (**oaA**) of less than or equal to 50% of the total follicles quantified (as per the definition of the WKMATCH [12]).
- Absence of post-ovulatory follicles (**POF**), beta atretic oocytes (**oaB**), oocytes at the end of vitellogenesis (**vit3**), oocytes in hydration (**pho**) and hydrated oocytes (**ho**).

The histological features to identify spawning (C) individuals were split into two for batch spawning species. The first description identified ovaries that were at the beginning of the spawning phase, stating that **vit3** oocytes are present, early stages of maturing oocytes can be present, atresia of vitellogenic or hydrating oocytes may be present, and there can be no **POF**. The classifying model takes into consideration the absence of **POF** but also adds the absence of beta atretic oocytes, further eliminating any slides that show signs of spawning. Moreover, the maturing follicles such as **vit3**, **pho** or **ho** are present, and alpha atretic oocytes must represent 50% or less of quantified follicles. The second histological feature description aims to identify ovaries at the end of the spawning phase. The ovaries are described as potentially having oocytes at the end of the germinal vesicle migration, oocytes with germinal vesicle breakdown, and oocytes in hydration or ovulation. Recently collapsed **POF** can be present. The model setup states that follicles such as **vit3**, **pho** or **ho** are present; alpha atretic oocytes represent 50% or less of oocytes quantified; beta atretic oocytes must be absent; and the number of **POF** does not exceed the number of **ho** quantified. The last two rules mark the limit between ovarian slides that are to be classified into the spawning (C) phase and those that are to be classified under the D (regressing/regenerating) phase. They allow for the presence of **POF**, but take into consideration that if there are more **POF** than hydrated oocytes, then the individual has finished spawning, especially if beta atretic oocytes are already present.

The histological features for omitted spawning individuals (E) are characterized by the absence of **POF** and with at least 50% of oocytes in alpha atresia (**oaA**). The model set up to classify the slides takes into consideration these two premises. To make sure that no slide is misclassified, the previously stated models for phases B and C include a rule that if a count of more than 50% of **oaA** is present, they are not to be classified into one of the previous stages, allowing them to be classified into phase **E**.

In regards to the regressing (Da) and regenerating (Db) phases, the Da phase is defined by the presence of atresia, **POF** and some cortical alveoli and/or **vit1** and **vit2** oocytes. The Db phase is characterized by the presence of solely oogonia and primary oocytes, with the potential presence of muscle bundles, enlarged blood vessels, thick ovarian walls, and/or atresia or old, degenerating **POF**.

The previously set up models do not intersect with one another, leaving any unclassified individuals to be categorized under the D phase. Setting up models to separate these individuals into two sub-phases would mean adding percentages to certain cellular structures or inferring a quality to the follicles, which would add subjectivity.

Finally, features such as the gonadal wall thickness, the intercellular space and the connective tissue were not implemented into the models. Indeed, with no definite possibility to quantify what is to be considered a “thin” or “thick” ovarian wall, or even what percentage of connective tissue, intercellular space or blood vessels is considered “scarce” or “a lot”, using this data would only have added subjectivity into our models. Moreover, these features could have led to a bias since the proportion they occupy on a cross section can be affected by the quality of the slide. Indeed, if the ovary has been nicked during the sampling, this could lead to missing tissues for the ovarian wall. A fresh ovary that has been stretched out during the extraction or during the cut into a smaller sample could show a lot of intercellular space. Finally, oocytes in the late stages of vitellogenesis tend to be ripped off the slide during the microtome cutting process. This leads to slides with missing oocytes that cannot be identified or the presence of “fake” intercellular space. The same problem also applies to connective tissue that, stretched out too thin at those later stages, also tends to be ripped away during the mounting process.

### 3.4. Histological and Macroscopic Maturity

By giving each of the 151 sampled fish a maturity phase through macroscopic criteria (1, 2, 3, 4) and a stereologically determined maturity (A, B, C, D, E), a confusion matrix (Table 4) was set up. The macroscopic method classified 94 fish in 1, 8 in 2, 3 fish in 3, and 46 in 4. The stereology method classified 23 fish in A, 53 in B, 9 in C, 65 in D and 1 fish in E. This table showed that for individuals classified as immature (1) with the macroscopic method, stereology placed 23.4% of individuals under that same phase (A), 47.9% into the developing (B) phase and 28.7% under the regressing/regenerating (D) phase. Individuals identified as developing with the macroscopic method (2) fell under either the spawning (C) or regressing/regenerating (D) phase when using stereology (87.5% and 12.5%, respectively). For the three individuals classified into the spawning phase with the macroscopic method, stereology confirmed the result for 2 individuals while placing the last one into the omitted spawning (E) phase. Finally, while the macroscopic method helped classify 80.4% of individuals correctly into the regressing/regenerating (D/4) phase, 2.2% and 7.4% of individuals were classified as immature (A) and developing (B), respectively, with the stereology method.

**Figure 4:**
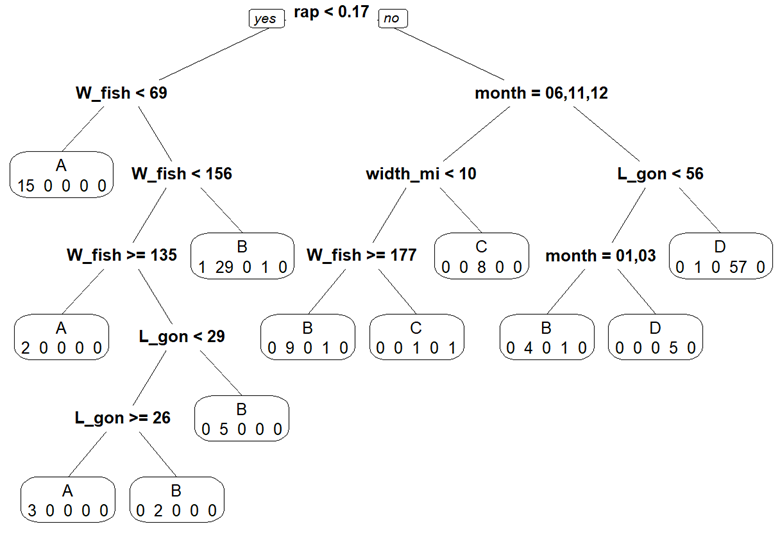
Classification tree with the identified maturity phase and the number of linked individuals categorized into that leaf, and with the measured parameter that separates the different leaves at the node. With the maturity phases determined through stereology (A, B, C, D, E), L_fish is the fish’s total length, W_fish is the fish’s ungutted weight, L_gon is the ventral ovary’s total length, month is the month the fish was sampled, and **rap** is the ratio between the ventral ovary’s length and the fish’s total length. Root node error = 81/146 (55%); *n* = 146.

The classification tree (Figure 4) has a root node error of 55.4% (*n* = 146). The first node is the ratio between the ventral ovary’s length and the fish’s total length (**rap** < 0.17). This first node separates all immature (A) individuals from all spawning (C) individuals. Individuals in the developing (B) phase are found throughout the entire tree. Individuals in the spawning (C) phase are found in months 11 (November) and 12 (December). However, all but one individual in the regressing/regenerating (D) phase are found when **rap** is greater than or equal to 0.17. The majority (85%) of individuals in phase D are found during the months 6 (June), 11 (November), and 12 (December) and for gonads with a length greater than or equal to 56 mm. The fish classified under omitted spawning (E) does not seem to have a macroscopic parameter that allows for it to be grouped by itself and instead was grouped under phase C.

**Figure 5:**
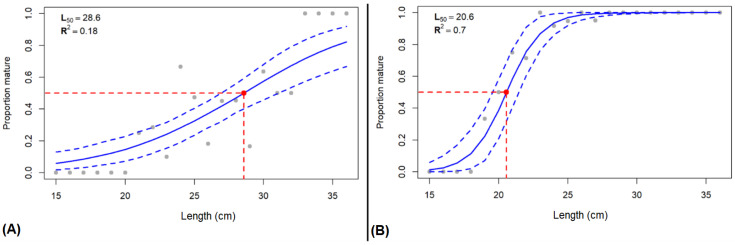
Maturity ogive and length at which 50% of the population has reached sexual maturity (L_50_, represented by the red dot) for female plaice collected in division VIId during the MATO project, from 2017 to 2019 (*n* = 151), calculated from (**A**) the macroscopic method (confidence intervals = 26.9–31.1 cm) and (**B**) the stereology method (confidence intervals = 19.6–21.5 cm).

Maturity ogive and the length at which 50% of the population has reached sexual maturity (L_50_) were determined for both data frames (Figure 5). For the visual maturity data, the L_50_ falls at 28.6 cm (R^2^ = 0.18). For the stereological maturity data, the L_50_ falls at 20.6 cm (R^2^ = 0.70).

## 4. Discussion

### 4.1. Maturity Phase Classification with Stereology

Histology is recognized as the most precise method to estimate sexual maturity through the detailed observation of ovarian sexual structures [16,51], with a cross section representing a snapshot in time for a single individual. Though we can infer the oogenesis development with enough sampling from individuals at different maturity phases, the identification of germline cells that are in transition between two stages can lead to difficulties in their identification. This may also lead to missing cellular stages if too much time passes between two samplings, especially if the species studied is in a maturity phase for a short time period. Another problem encountered, linked to the fact that sampling relied on the catch of the day from the nearby fish auction house, was the limited access to immature individuals, which was necessary to calculate a maturity ogive. This led to a size-targeted sampling (August 2019) for small-sized plaice to ensure that immature individuals were sampled and a size at which 50% of the population has reached sexual maturity (L_50_) could be calculated. While all individuals were from the same stock, we could not test if immature individuals taken during the first sampling events would have yielded L_50_ different results.

Using stereology means the ratio of structures quantified are from a random sampling grid. This assures for a more objective data on what structures are present or absent. With an adapted grid, the results will be reliable and allow classification of the individual into a maturity phase. In our case, it was shown that very small structures such as oogonia may be underrepresented; however, this has little to no impact on the classification model thereafter since primary oocytes are reliably identified. With the difference in sizes between oogonia and vitellogenic oocytes, if one wanted to enhance the precision to quantify these smaller structures, then they could add more points to the counting grid or manually count all oocytes. Moreover, this study set up a method to check for cellular homogeneity, testing that the position of the cross section within the ovary will not influence the ratio of quantified structures for individuals sampled at the beginning of the spawning period [35]. In this current study the cellular homogeneity was not tested for individuals in other maturity phases. Using only the median section of the oocyte could be a bias if this homogeneity is not kept at the appearance of cortical alveoli oocytes, at the appearance of hydrating or hydrated oocytes, or during the spawning. However, the plaice is considered a batch spawner with a determinate fecundity (no new oocytes will appear before the end of the spawning season), and the oocyte development is homogeneous at the beginning of the spawning period, with sexually mature individuals presenting cortical alveoli and vitellogenic oocytes. It was thus accepted that this homogeneity is kept throughout the cycle.

The quantitative histology-based model set up for the classification of individuals into a maturity phase follows ICES criteria [11], which does take into account species that are batch spawners like *Pleuronectes platessa* [28] (a portion of eggs are released at different intervals during the spawning season). Moreover, this species has a determinate fecundity (total fecundity prior to the onset of spawning is considered to be the potential annual fecundity) with a group synchronous oocyte development (at least two populations of oocytes at any one time) [13]. This means that multiple oocyte stages will be present during the entire oogenesis cycle. The study of the oogenesis cycle of the species before classifying these individuals into a maturity phase is primordial since it will greatly influence the rules set to classify the individuals into maturity phases. Since an identification of the two spawning (C) sub-phases would have required the implementation of thresholds, the model presented in this paper identifies individuals that are either at the beginning of their first spawning event, or that have already spawned at least once and are between two spawning events or spawning again. Whatever their sub-phases, they are all classified under C. Finally, even if the quantitative histology-based model was generalized as much as possible, thresholds on certain criteria had to be implemented (the portion of germline cells is greater than or equal to 50% of the portion of atretic oocytes alpha to identify omitted spawning individuals).

Phase D (regressing/regenerating) could not be modeled because of the complexity of present structures within the ovary at this phase. Indeed, the presence of post-ovulatory follicles (**POF**) at different stages of degeneration, the quantity of atretic oocytes (still in their theca or that are in the ovarian lumen), the presence of oocytes that may undergo atresia at a later time, the development of a new oocyte cohort for a future spawning season or the presence of oocytes that will mature during a future spawning season are all criteria that must be taken into account when classifying individuals by the presence or absence of their germline cells [10]. These criteria are species dependent and will greatly change as the ovary goes through the regressing/regenerating phase, making the implementation of a model to identify these individuals complex, if not impossible, when making sure that there are no redundancies with the rules set up for the classification into other maturity phases. Though other criteria such as gonadal wall thickness, blood vessel ratio or spatial volume of the lumen could be added, it is important to keep in mind that they must be quantified through stereology to reduce the subjective aspect in their identification and once again are very species specific.

### 4.2. Inter-Rater Reliability

In 2006 [23], it was found that a bias could be found when field agents identified the sexual maturity of female plaice using only the macroscopic method. Females that had spawned (D) were assigned with the highest consistency, while there was disagreement for immature (A) and developing (B) females. When comparing the macroscopic results with the stereology ones from the current study, similar results are found. The best identified phase is D (80.4%), while no individuals were correctly identified under B. Indeed, 47.9% of individuals that were identified as immature (A) were actually in development (B), while 87.5% classified under B were actually in C.

There is a clear difficulty when determining the maturity phase through either the observation of macroscopic criteria of the ovary, or individual parameters such as body length or weight. When classifying individuals by their biological parameters, depending on their maturity phase determined through the stereological method (Figure 4), only immature (A) and spawning (C) individuals were completely separated by the ratio between the ventral ovary’s length and the fish’s total length. No clear patterns could be extracted.

For the stereological readings, while the inter-rater reliability was estimated with a percentage agreement and Fleiss’ kappa, the first readings were not recorded, so the evolution of the reading method before and after the reading protocol was set up could not be compared, thus limiting our view of the impact of the reading protocol on those results. However, even if the pre-calibration results were not available for this study, a similar exercise was made for the striped red mullet (*Mullus surmuletus*), showing that the implementation of guidelines could help improve the reading agreement percentage up to 25%, with the lowest agreement percentage being 68% for the final read (personal observations to be published at a later date). This should underline the importance of a calibration exercise before the readings, as well as setting up a reading protocol to limit interpretations during the identification of structures.

### 4.3. Impact of Macroscopic Versus Stereology Methods

When estimating a maturity ogive for this species, the macroscopic method yielded an L_50_ of 28.6 cm. When using the stereological method, the L_50_ is 20.6 cm. This difference in estimations is of great importance for multiple reasons. The first reason is the importance of accurate maturity data since it will be used to estimate factors such as maturity ogive, the length/age at first maturity or the Spawning Stock Biomass. Values that will impact stock assessment models and thus could lead to biases in management-related metrics, like when implementing a minimum landing size (MLS). Currently, the MLS for *Pleuronectes platessa* is set at 27.0 cm [52], which means that if we used the macroscopic L_50_ at 28.6 cm, then not even half of individuals caught at that length have participated in at least one spawning season. However, if we go by the stereological L_50_, then individuals caught at 27 cm have surely participated in at least one spawning season. For this species, the macroscopic maturity favors catching bigger individuals that are sure to have already reproduced; however, this study greatly underlines the limitations of the macroscopic method and raises the question about the reliability of data collected for other species. An accurate and standardized reading on *P. platessa*’s maturity guarantees a high data reliability, which is important when following a stock, especially since it has been shown that the L_50_ for North Sea plaice has declined over the past half century [53,54].

## 5. Conclusions

Stereology on histological ovarian cross sections, coupled with a calibration exercise and standardized reading procedure, allows for a more objective identification of the sexual maturity phase when compared to the macroscopic method, while being just as precise as the histological method. Once the cellular structures have been quantified, the individual can be classified into a sexual maturity phase, though the model setup will greatly vary on the gametogenesis development of the species. Even if this method requires a lot more resources compared to the more classical macroscopic method, and more time when reading the histological slides, it clearly shows differences in data output and ultimately impacts fisheries assessment indexes such as maturity ogives.

## Figures and Tables

**Table 1:** Summary for sampled individuals from the English Channel stock (ICES division VIId).

Month	Year	Number of Fish Sampled
January	2017	5
December	2017	5
March	2018	10
June	2018	12
November	2018	14
December	2018	10
January	2019	24
February	2019	23
March	2019	30
August	2019	18

**Table 2:** List of the 19 cellular structures, and their abbreviations, used for the stereology reading of the ovarian histological slides for *P. platessa*. * = main germline cells.

Abbreviation	Name
og *	oogonium
po1 *	primary oocyte, early stage
po2 *	primary oocyte, late stage
cao *	cortical alveoli oocyte
vit1 *	oocyte in early vitellogenesis
vit2 *	oocyte in vitellogenesis with *zona radiata* growth
vit3 *	oocyte at the end of vitellogenesis
pho *	partially hydrated oocyte
ho *	hydrated oocyte
POF *	post-ovulatory follicle
aoA *	atretic oocyte alpha
aoB *	atretic oocyte beta
L	lysis
cs	blood vessel
ei	intercellular space
pg	gonadal wall
tc	connective tissue
v	unnatural emptiness
i	undetermined

**Table 3:** For all 20 slides, percentage agreement (Agree (%)) and Fleiss’ kappa (%) between 2 or 3 readers. With the Agree (%) for 3 or more readers and Fleiss’ kappa for n readers. With Slide ID the identification tag given to the ovarian section that was read under stereology.

Slide ID	Number of Raters	Agree (%)	Fleiss’ Kappa (%)
P 230117 7D 22 88 F1	3	71.9	73.7
P 230117 7D 19 72 F1	3	77.7	79.9
P 210117 7D 23 130 F1	3	76.6	78.5
P 110618 7D 31 297 F2A V2	3	83.3	85.2
P 110618 7D 30 257 F4 V2	3	87.2	88.2
P 110618 7D 30 243 F2A V2	3	76.2	79.1
P 110618 7D 29 237 F4 V2	3	87.8	89.0
P 110618 7D 28 249 F2A V2	3	73.4	76.4
P 110618 7D 28 227 F2A V2	3	69.0	71.9
P 110618 7D 27 225 F4 V2	3	85.7	86.6
P 110618 7D 27 215 F4 V2	3	81.6	83.6
P 110618 7D 27 206 F2A V2	3	79.8	82.0
P 110618 7D 28 247 F4 V2	2	*NA*	82.9
P 220117 7D 23 116 F1	2	*NA*	77.7
P 230117 7D 21 80 F1	2	*NA*	79.8
P 150318 7D 36 523 F4 V2	2	*NA*	83.0
P 150318 7D 31 287 F4 V2	2	*NA*	84.9
P 150318 7D 30 302 F4 V2	2	*NA*	82.7
P 150318 7D 30 271 F4 V2	2	*NA*	83.7
P 150318 7D 29 282 F2A V2	2	*NA*	74.5

**Table 4:** Confusion matrix for all 151 female plaice (*P. platessa*) classified into a maturity phase with the macroscopic method (1, 2, 3, 4) and a stereologically determined maturity phase (A, B, C, D, E). With immature (A & 1), developing (B & 2), spawning (C & 3), regression/regeneration (D & 4) and omitted spawning (E).

	1	2	3	4
A	22	0	0	1
B	45	0	0	8
C	0	7	2	0
D	27	1	0	37
E	0	0	1	0

## Data Availability

All data used in the following article is available on the Zenodo repository (http://doi.org/10.5281/zenodo.3745640). R Scripts are available by contacting Carine Sauger (carine.sauger@gmail.com) and Laurent Dubroca (laurent.dubroca@gmail.com).

## Supplementary Materials

The following supporting information can be downloaded at https://www.mdpi.com/article/10.3390/ani16030519/s1, Table S1: Function *drop1* outputs of the generalized linear models (GLM binomial, logit link) for each histological structure found throughout the 90 slides. With **slide** the identification number of the sampled fish (*n* = 15), **position** the section’s position within the ovary (*n* = 6), **df** the degrees of freedom, deviance the residual deviance of the model, **AIC** the Akaike information criterion and **P(Chi)** the *p*-value calculated under a Chi^2^ distribution, Table S2: Terminologies used by the ICES for maturity staging, and descriptions of the sexual state, maturity phase, macroscopic and histological criteria of teleosts. Based on the definitions and descriptions of the ICES (WKMATCH [12] & WKASMSF [11]) and Brown-Peterson et al. [10].
