# Supplementary material for: Comparing Macroscopic and Quantitative Histological Methods to Determine Sexual Maturity in the Female European Plaice, Pleuronectes platessa Linnaeus, 1758
Source: Animals (Basel). 2026 Feb 6;16(3):519. doi: 10.3390/ani16030519 (PMC12896940; doi:10.3390/ani16030519)
Supplement: Supplementary file 1 [file animals-16-00519-s001.zip › Table S1.pdf]

		df	deviance	AIC	P (Chi)
**ov**	Intercept		32,40	98,99	
	slide	14	53,25	91,83	0.10
	position	5	33,92	90,51	0.91
**op1**	Intercept		156,39	341,19	
	slide	14	194,49	351,29	<0.001 ***
	position	5	174,38	349,18	0.003 **
**op2**	Intercept		420,64	910,90	
	slide	14	1307,73	1770,00	<0.001 ***
	position	5	483,85	964,10	<0.001 ***
**oca**	Intercept		215,82	616,57	
	slide	14	1325,99	1698,74	<0.001 ***
	position	5	234,33	625,09	0.002 **
**vit1**	Intercept		175,70	404,01	
	slide	14	4552,28	4752,59	<0.001 ***
	position	5	205,08	423,38	<0.001 ***
**vit2**	Intercept		21,67	101,64	
	slide	14	906,80	958,78	<0.001 ***
	position	5	42,65	112,62	<0.001 ***
**L**	Intercept		443,87	723,92	
	slide	14	668,75	920,80	<0.001 ***
	position	5	595,39	865,45	<0.001 ***
**oaA**	Intercept		51,51	124,06	
	slide	14	154,28	198,84	<0.001 ***
	position	5	80,29	142,84	<0.001 ***
**oaB**	Intercept		7,12	70,24	
	slide	14	855,88	891,01	<0.001 ***
	position	5	318,13	371,25	<0.001 ***
**POF**	Intercept		9,21	76,27	
	slide	14	229,89	268,95	<0.001 ***
	position	5	18,05	75,11	0.11
**pg**	Intercept		678,17	1269,09	
	slide	14	3317,77	3880,69	<0.001 ***
	position	5	938,02	1518,94	<0.001 ***
**tc**	Intercept		435,66	1013,90	
	slide	14	566,28	1116,52	<0.001 ***
	position	5	517,81	1086,05	<0.001 ***
**cs**	Intercept		142,03	426,52	
	slide	14	284,18	540,67	<0.001 ***
	position	5	156,74	431,23	0.01 *
**ei**	Intercept		754,39	1387,79	
	slide	14	1269,55	1874,94	<0.001 ***
	position	5	825,42	1448,81	<0.001 ***
**i**	Intercept		39482,00	40850,00	
	slide	14	40046,00	41386,00	<0.001 ***
	position	5	39523,00	40881,00	<0.001 ***
**v**	Intercept		6636,40	7280,70	
	slide	14	9190,80	9807,10	<0.001 ***
	position	5	6694,80	7329,50	<0.001 ***
