# Supplementary material for: Comparing Macroscopic and Quantitative Histological Methods to Determine Sexual Maturity in the Female European Plaice, Pleuronectes platessa Linnaeus, 1758
Source: Animals (Basel). 2026 Feb 6;16(3):519. doi: 10.3390/ani16030519 (PMC12896940; doi:10.3390/ani16030519)
Supplement: Supplementary file 1 [file animals-16-00519-s001.zip › Table S2.pdf]

State description	Phase		Old terminology (ICES 2012)		New terminology (ICES 2018)		Macroscopic criteria		Histological features		Histological model
		Possible sub-phase		Possible sub-phase		Possible sub-phase		Possible sub-phase		Possible sub-phase	
SI : Sexually immature, (without gonad development)	Immature		I		A		Small pinkish and translucent (often clear) ovaries shoter than 1/3 of body cavity. Indistinct blood vessels and no visible eggs		Only oogonia and PG oocytes present containing no oil droplets, rare atresia, no muscle bundles. Thin ovarian wall, scarce connective tissue around follicles and little space between oocytes		Presence of either **og**, **po1** or **po2** cells<br>Absence of **cao**, **vit1**, **vit2**, **vit3**, **pho**, **ho** and **POF**
SM : Sexually mature (with gonad development)	Developping	Developing but functionally immature (first-time developer)	II	IIa	B	Ba	Enlarging ovaries, blood vessels become more distinct	Small pinkish/reddish ovaries shorter than 1/2 of the body cavity. Eggs not visible to the naked eye	Marked increase in oocyte size, blood vessels become more distincts, PG, CA oocytes present. Vtg1 and Vtg2 oocytes can also be present. No POF. No Vtg3 oocytes. Some atresia may be present.		Presence of either **cao**, **vit1** or **vit2** cells<br>Absence of **vit3**, **pho**, **ho**, **aoB** and **POF**<br>A percentage count of **aoA** of less than or equal to 50% of the total follicles quantified
		Developing but functionally mature		IIb		Bb		Pinkish-reddish/reddish-orange and translucent ovaries that have a length of about 1/2 of the body cavity. Visible blood vessels, no visible eggs			
	Spawning	Actively spawning	III	IIIa	C	Ca	Large ovaries, blood vessels prominent, individual oocytes visible macroscopically	Pinkish-yellow ovaries with a granular appearance and a length of about 2/3 of the body cavity. Eggs are visible to the naked eye through the *ovaric tunica* which is not yet translucent. No eggs are expelled under light pressure		Oocytes at the end of GVM, GVBD, hydration or ovulation are present. Recently collapsed POF can be present	**Ca** :<br>Presence of either **vit3**, **pho** or **ho** cells<br>Absence of **aoB**<br>A percentage count of **aoA** of less than or equal to 50% of the total follicles quantified<br>A percentage of **POF** that does not exceed the number of **ho**
		Spawning capable		IIIb		Cb		Orange-pink ovaries with conspicuous superficial blodd vessels and a length from 2/3 to full length of the body cavity. Large, transparent, ripe eggs are clearly visible and can be expelled under light pressure, or escape freely		Vtg3 oocytes are present. Early stage of OM can be present. No POF. Atresia of vitellogenic or hydrating oocytes may be present	**Cb** :<br>Presence of either **vit3**, **pho** or **ho** cells<br>Absence of **POF** and **aoB**<br>A percentage count of **aoA** of less than or equal to 50% of the total follicles quantified
	Regression / Regeneration	Regression	IV	VIa	D	Da		Reddish ovaries of about 1/2 of the body cavity length. Flaccid ovarie walls, prominent blood vessels, and possible remnants of disintegrating opaque and/or translucent eggs		Atresia (any stage) and POF are present. Some healthy CA and/or Vtg1, Vtg2 oocytes present	
		Regeneration		IVb		Db		Small pinkish and translucent ovaries that have a length of aboiut 1/3 of the body cavity, with reduced but present blood vessels. No visible eggs		Only oogonia and PG oocytes present. Oil droplets can be seen in PG oocytes (species dependent). Muscle bundles, enlarged blood vessels, thick ovarian wall and/or atresia or old, degenerating POF may be present. There is more space and interstitial tissues around PG oocytes	
	Omitted spawning		V		E				No POF and at least 50% of the vitellogenic oocytes are atretic		Abscence of **POF**<br>A percentage count of **aoA** of more than 50% of the total follicles quantified
	Abnormal		VI		F		Problems in the gonad development (necrosis, sclerosis, intersex, majority of the gonad that looks unhealthy)				
